# The impact of health risk communication on self-perceived health and worry of targeted groups: Lessons from the Swedish COVID-19 response

**DOI:** 10.1371/journal.pone.0311596

**Published:** 2025-01-17

**Authors:** Jonna Rickardsson, Charlotta Mellander

**Affiliations:** Department of Economics, Centre for Entrepreneurship and Spatial Economics (CEnSE), Jönköping International Business School, Jönköping, Sweden; Wollega University, ETHIOPIA

## Abstract

**Background:**

The Swedish COVID-19 strategy aimed to protect vulnerable groups through targeted measures, categorizing individuals aged 70 and above as high-risk. This study examines the impact of such group-based risk assessments on subjective health and virus-related concerns among older adults.

**Methods:**

We analyzed survey data from the SOM Institute for 68- to 71-year-olds in 2019 (N = 684) and 2020 (N = 726). Using ordered logit regression, we compared perceived health and virus-related concerns between individuals just below (68–69 years) and just above (70–71 years) the high-risk age threshold, controlling for demographic factors.

**Results:**

In 2020, 70-year-olds reported significantly lower perceived health compared to their 69-year-old peers, a difference not observed in 2019. Furthermore, 70-year-olds exhibited significantly higher virus-related concerns than their slightly younger counterparts. These patterns persisted when expanding the analysis to include individuals aged 68 and 71.

**Conclusion:**

Our findings suggest that the Swedish COVID-19 strategy, while aimed at protecting individuals aged 70 and over, may have inadvertently increased perceived vulnerability and health concerns within this group. Conversely, those just below the high-risk age threshold reported better health and lower virus-related concerns, highlighting potential unintended psychological consequences of age-based risk communication.

## Introduction

In March 2020, the COVID-19 pandemic spread globally, prompting policymakers worldwide to quickly implement various measures to mitigate its adverse effects. As it became apparent that older individuals were particularly vulnerable to severe COVID-19 infection [1–3], many countries introduced lockdowns and social distancing measures for this group [4]. Sweden adopted a unique strategy for managing the COVID-19 pandemic, relying primarily on recommendations rather than mandates. However, like many other nations, Sweden’s strongest recommendations were directed at individuals aged 70 and older [5, 6]. Starting in March 2020, the Public Health Agency of Sweden specifically advised this age-group to minimize close contact with others as much as possible.

During the following critical months, individuals aged 70 and above were advised to exercise particular caution, avoid social gatherings, and stay away from crowded places. The public was also urged to take measures to protect individuals in this age group, along with others at greater risk of severe illness. Nursing homes implemented visitor bans and introduced new hygiene protocols to safeguard older adults. In media, statistics on ICU admissions and deaths consistently emphasized the 70-and-over age group. Public authorities thus categorized everyone aged 70 and above as a single vulnerable group, often overlooking other risk factors, such as underlying health conditions which often correlate with age. The term "over 70" was widely used by health authorities and the media to describe one of the most vulnerable groups in society. The age-specific recommendations remained in place until the end of October 2020, when they were lifted—not because the risk of COVID-19 had decreased, but due to concerns that prolonged isolation had worsened mental health among the targeted groups. A report from the Public Health Agency highlighted several negative consequences, including social isolation, frustration, and the perceived stigmatization and special treatment of those classified in the high-risk group [7].

Previous research also suggests that this narrative contributed to increased ageism and stigma [8, 9], which may have exacerbated well-being challenges among older adults [10, 11]. In addition to identifying age as a key risk factor for severe COVID-19 outcomes, further studies have highlighted the broader negative effects of the pandemic on older individuals’ well-being [12, 13]. However, limited attention has been given to the potentially added health implications stemming from older adults’ self-perception as part of a high-risk group, shaped by group-based risk assessments and public health messaging. This study seeks to fill this gap by examining how age-specific communication affects individuals’ perceived health status and their concerns related to COVID-19. We hypothesize that continuous reminders of one’s high-risk status may negatively influence perceived health and heighten concerns about the virus.

Specifically, we investigate whether being categorized as part of a "high-risk group" lowered individuals’ perceptions of their general health and amplified their concerns about COVID-19, focusing on those who recently entered the high-risk category (age 70–71), compared to individuals just 1–2 years younger. We examine perceived health in this age span both in 2019 and 2020. Our identification strategy relies on the assumption that individuals just below age 70 are similar to those just above, except for their risk-group classification. Any observed differences in perceived health between these groups in 2020, but not in 2019, are thus likely due to differing pandemic recommendations and risk-group assessments—along with the associated factors of isolation, stigma, and ageism.

While factual dissemination is crucial during a pandemic, the way information is communicated may also play a vital role. Effective health risk communication depends on various factors, including cultural and social contexts, and attitudes toward public health interventions [14]. While research often suggests that effective disease communication should rely on the transmission of facts through proper channels, it is also important to note that even factual messages can be perceived and interpreted differently by different groups of individuals [15]. Factual dissemination through government messaging is a key tool for influencing public views and behaviors during crises and well-considered government communication can help reduce harm [16]. However, framing a specific group, such as older individuals, as “old and vulnerable” may contribute to negative social identities, increase ageism, and elevate fear and anxiety [8, 9, 17]. Furthermore, focusing solely on the risks to older adults may fail to significantly improve attitudes or behaviors in the broader population and could even be counterproductive, as younger individuals might perceive themselves as completely safe. In contrast, when younger adults receive information about the risks posed to their age group, they are more likely to see the disease as a threat and adhere to public health recommendations [18].

Relatedly, several studies have explored the role of age in adherence to COVID-19 recommendations during the early stages of the pandemic. One study found that while older adults were less likely to use public transportation or attend social gatherings, they were not consistently more willing to self-isolate or wear face masks [4]. In contrast, other research shows a positive correlation between age and overall compliance with public health measures. In France, older individuals were more likely to follow guidelines, possibly due to heightened vulnerability [19]. In the U.S., older adults were significantly more likely to perceive the pandemic as a "significant crisis" and a "threat to health" compared to younger Americans [20]. This heightened perception of risk may explain why subsequent research in Italy and the U.S. found that older adults were especially likely to adhere to health measures and reduce social interactions [21, 22].

Nudges in the form of reminders often play a role in health communication. One study concluded that dentist check-up reminders more than doubled the percentage of patients who made an appointment [23]. However, another study found that while nudges during the COVID-19 pandemic may have influenced intentions, they did not always translate into actions. Only individuals with poorer health status stayed home more after receiving a reminder, whereas those in good health did not significantly change their behavior [24].

Overall, it remains unclear whether age-specific recommendations and reminders prompted individuals classified as high-risk to behave differently than those just 1–2 years younger and not yet in the high-risk category. However, the findings from this study suggest that such age-specific communication led 70-year-olds in 2020 to perceive their general health as significantly worse compared to their slightly younger peers, a distinction that did not exist prior to the pandemic. Additionally, 70-year-olds expressed significantly greater concern about the virus than those aged 1–2 years younger. These findings highlight the potential unintended consequences of age-specific communication strategies during the COVID-19 pandemic.

While this study does not evaluate the effectiveness of Sweden’s pandemic communication strategy in protecting the elderly from infection or mortality (as it does not examine the impact of age-specific communication on these outcomes), our findings emphasize the importance of considering the broader, potentially unintended consequences of group-specific recommendations. By exploring the effects of age-targeted communication on perceived health status, this study contributes to the ongoing discussion on the development of effective risk communication strategies during public health crises.

## Methods

In this study, we utilize survey data from the "National SOM" survey for the years 2019 (before the pandemic) and 2020 (during the pandemic) [25, 26]. The SOM Institute, or the Institute for Opinion Surveys and Media Analysis, is a research institute based at the University of Gothenburg in Sweden, and its yearly National SOM survey has been conducted since 1986. The survey aims to provide a comprehensive understanding of Swedish society by collecting data on a wide range of topics, including social issues and values. The survey involves a large, random, and representative sample of the Swedish population aged 16–85 years. Questionnaires are sent out in September each year and the fieldwork is completed 3–4 months later (December/January). The survey response rate was 49 percent, and 51 percent in 2019 and 2020 respectively. The SOM Institute adheres to rigorous methodological standards in survey design, sampling, and data analysis to ensure quality. Yearly methodology reports compare the sample with the overall Swedish population for representativeness of the data. According to the reports, foreign-born individuals, younger individuals, and men (especially younger men) are somewhat less likely to respond to the survey than older individuals, Swedish born individuals, and women [27, 28].

To assess the direct effects of age-specific communication on self-perceived health and concern for the COVID-19 virus, we use a restricted sample from the survey that includes only individuals aged 68–71. In this age group, the response rate is even higher, and the skewness based on age and gender is negligible. In 2020, the sample of 68-71-year-olds consists of 726 individuals (Mean = 69.478, Sd = 1.094). In 2019, the sample of 68-71-year-olds totals 684 individuals (Mean = 69.575, Sd = 1.137).

To address our research question regarding the distinct effects of targeted information aimed at a specific age group on perceived health, we analyze individuals’ self-reported health both before and during the pandemic. We operate under the assumption that individuals aged 68–69 and 70–71, on average, are similar in most aspects except for their risk-group classification. Consequently, any disparities in health and COVID-19 related concerns observed between individuals just below and just above 70 in 2020, but not in 2019, can likely be attributed to their risk group belonging. These disparities may be influenced by varying media portrayals, communicated recommendations, worsening ageism, or perceived vulnerability.

We utilize the following survey questions as dependent variables: *How would you rate your general health*? with 10 response options ranging from 1 = Very bad to 10 = Very good (2019: Mean = 7.633, Sd = 1.978; 2020: Mean = 7.494, Sd = 2.124), and *How worried are you about the coronavirus and its consequences for*: *a) yourself*, *b) your close relatives and friends*, *c) the Swedish society*, with the four response options; 1. Not at all worried, 2. Not very worried, 3. Quite worried, and 4. Very worried.

Our main variable of interest is the age of the respondent, i.e., whether the respondent is just below 70, or 70 and above, as we examine potential differences between 69- and 70-year-olds (and 68-69- and 70-71-year-olds) in their self-reported health perception before and during the pandemic.

We also have access to information about the individual and/or household characteristics. We add a set of control variables in our analysis, in accordance with previous research [10, 29]. These control variables include household income, higher education, gender, type of housing, marital status, place of residence, and month of response. We control for the urban-rural categorization of the place of residence, which includes rural area, smaller agglomeration, city or larger agglomeration, and the three largest cities/metropolitan areas of Stockholm, Gothenburg, and Malmo. We also control for the month of response, which ranges from September to December/January. The summary statistics for the dependent and independent variables are presented in S1 Table in S1 File.

Various methodological approaches can be used to analyze subjective health and coronavirus concerns. In this study we use quantitative methods. This is a common approach in previous research [10, 11, 29]. We first compute descriptive statistics and compare age groups using t-tests for the variables of interest, both for 2019 and 2020. We then run regression analyses where we include a set of control variables in our analyses to address potential confounding factors and increase the robustness of our findings. Since the survey samples include different individuals in 2019 and 2020, we conduct cross-sectional analyses. Given that the dependent variables are of a categorical and ordinal nature, we utilize ordered logit regressions to estimate the relationships of interest. This approach enables us to utilize all response categories of the dependent variables.

The ordered logit model uses a Maximum Likelihood method to estimate the probability that an individual chooses a higher health or concern response option as a function of the independent variables. It estimates the likelihood that an individual will cross a threshold. The ordered logit model does not require the dependent variable to be continuous, normally distributed, or to have a linear relationship with the independent variables [30].

All statistical analyses were conducted using Stata 18. The ordered logit regressions were estimated using the ologit command, with odds ratios presented to aid interpretation–thus values greater than 1 indicate a positive relationship, and values less than 1 indicate a negative relationship.

## Results

Tables 1 and 2 present the results of t-tests comparing the mean values of individuals aged 69 and 70, as well as 68–69 and 70–71, for the variables "Subjective health status" and "Coronavirus concerns" regarding oneself, family and friends, and society. Table 1 focuses on the comparison between 69- and 70-year-olds, while Table 2 broadens the comparison to include 68-69- and 70-71-year-olds. Naturally, data on coronavirus-related concerns are only available for 2020 due to the pandemic context.

**Table 1:** Comparison of 69- and 70-year-olds (mean values) in 2019 and 2020.

		2020			2019	
	Age: 69	Age: 70	*p-value*	Age: 69	Age: 70	*p-value*
Subjective health	7.85	7.42	0.034	7.75	7.50	0.237
Corona virus concerns for:						
•oneself	2.50	2.79	0.000			
•family and friends	2.88	3.03	0.053			
•the society	3.01	3.06	0.533			
*N*	203	180		163	163	

**Table 2:** Comparison of 68-69- and 70-71-year-olds (mean values) in 2019 and 2020.

		2020			2019	
	Age: 68–69	Age: 70–71	*p-value*	Age: 68–69	Age: 70–71	*p-value*
Subjective health	7.70	7.27	0.006	7.71	7.57	0.348
Corona virus concerns for:						
•oneself	2.57	2.78	0.000			
•family and friends	2.89	3.02	0.025			
•the society	2.99	3.04	0.422			
*N*	376	350		325	359	

In 2020, the difference in subjective health between individuals aged 69 and 70 (as well as 68–69 and 70–71) is statistically significant. Specifically, those aged 70(-71) perceived their health status as significantly worse than those under 70—a distinction that was not present in 2019, before the pandemic.

A similar pattern is observed for "Concern about the coronavirus and its consequences for oneself." Individuals aged 70(-71) expressed significantly greater concern compared to their slightly younger counterparts. While concerns for family and friends also differed significantly between the groups, the significance level was lower. No significant differences were found between the groups regarding concerns for society as a whole.

Thus, the descriptive statistics in Tables 1 and 2 reveal significant differences in self-reported health status and concern for the coronavirus between individuals aged 69 and 70 (as well as 68–69 and 70–71) in 2020. Specifically, those aged 70(-71) reported significantly worse health status and higher levels of concern about the virus compared to those aged 69(-68). These findings suggest that communication targeted at this age group may have influenced their health perceptions and concerns regarding the virus.

Table 3 presents the results from the ordered logit estimations. We conducted separate regressions for the years 2019 and 2020 to assess potential changes in subjective health before and during the pandemic, controlling for other factors. The first two columns (1 and 2) illustrate differences between individuals aged 69 and 70, while the last two columns (3 and 4) compare the groups aged 68–69 and 70–71.

**Table 3:** Subjective health among individuals aged 69–70 and 68–71 in 2019 and 2020.

	Age: 69–70		Age: 68–71	
	2019	2020	2019	2020
	(1)	(2)	(3)	(4)
Age 69: ref. category				
Age 70	0.994	0.732*		
	(0.203)	(0.136)		
Age 68–69: ref. category				
Age 70–71			0.972	0.724**
			(0.133)	(0.097)
Household income	1.161***	1.131***	1.113***	1.169***
	(0.055)	(0.045)	(0.034)	(0.036)
Higher education	1.465*	1.264	1.416**	1.106
	(0.335)	(0.253)	(0.220)	(0.162)
Female	2.186***	1.327	1.689***	1.524***
	(0.467)	(0.260)	(0.243)	(0.215)
Villa/ townhouse	0.788	1.065	1.482**	1.356*
	(0.202)	(0.241)	(0.245)	(0.218)
Single: ref. category				
In a relationship	0.585	0.981	0.628	0.960
	(0.317)	(0.528)	(0.241)	(0.354)
Cohabitation	1.141	0.944	0.898	1.039
	(0.495)	(0.344)	(0.256)	(0.264)
Married/ Registered partnership	0.751	1.012	0.763	0.991
	(0.260)	(0.306)	(0.173)	(0.204)
Widow/ Widower	0.812	0.950	0.623	1.487
	(0.407)	(0.548)	(0.217)	(0.559)
Rural area: ref. category				
Smaller agglomeration	1.114	0.768	1.487*	1.124
	(0.363)	(0.235)	(0.336)	(0.241)
City or larger agglomeration	1.188	0.815	1.540**	0.859
	(0.355)	(0.213)	(0.312)	(0.170)
Stockholm/ Goteborg/ Malmo	0.939	0.766	2.114***	0.912
	(0.374)	(0.279)	(0.569)	(0.244)
Month: September: ref. category				
Month: October	1.394	0.686*	1.139	0.835
	(0.307)	(0.149)	(0.169)	(0.130)
Month: November	1.786	1.386	1.273	1.096
	(0.813)	(0.635)	(0.426)	(0.353)
Month: Dec/Jan	3.442	0.263	0.803	0.949
	(2.822)	(0.395)	(0.425)	(0.515)
Cut off points	Yes	Yes	Yes	Yes
Pseudo *R*^2^	0.030	0.019	0.022	0.022
N	326	383	684	726

Notes: Ordered logit regression. Coefficients displayed as odds ratios. Dependent variable: ’How would you assess your health in general?’ were 0 = Very bad and 10 = Very good. Standard errors in parentheses.

* p<0.10

** p<0.05

*** p<0.01

In 2019, no significant differences were observed in subjective health status between individuals aged 69 and 70 (column 1) or between those aged 68–69 and 70–71 (column 3). The coefficients for both comparisons were close to 1 (0.994 and 0.972, respectively) and not statistically significant. However, in 2020, the older age group, identified by the Swedish Public Health Agency as particularly vulnerable, reported significantly lower subjective health compared to those a year younger who were not specifically targeted by health authorities. The coefficients, 0.732 and 0.724, indicate a notably lower perceived health status in the older age group (columns 2 and 4).

To explore whether there were also significant differences in concerns about the coronavirus between the two age groups, we conducted an ordered logit regression using concerns about the coronavirus (for oneself, family and friends, and society) as the dependent variable. Since this variable is only available for 2020, we limited our analysis to data from that year. The results, expressed as odds ratios, are presented in Tables 4 and 5 for the two age group comparisons (69 vs. 70 and 68–69 vs. 70–71). The complete S2 and S3 Tables are available in the S1 File.

**Table 4:** Concern about the coronavirus among individuals aged 69–70, 2020.

	Concern for oneself	Concern for family and friends	Concern for society
	(1)	(2)	(3)
Age 69: ref. category			
Age 70	2.053***	1.542**	1.119
	(0.414)	(0.309)	(0.225)
Control variables:	Yes	Yes	Yes
Cut off points	Yes	Yes	Yes
Pseudo *R*^2^	0.056	0.065	0.056
N	383	383	383

Notes: Ordered logit regression. Coefficients displayed as odds ratios. Dependent variables: ’How worried are you about the coronavirus and its consequences for: 1) Yourself? 2) Your family and friends? 3) Society?’ were 1 = Not at all worried, 2 = Not quite worried, 3 = Somewhat worried, and 4 = Very worried. Standard errors in parentheses.

* p<0.10

** p<0.05

*** p<0.01

**Table 5:** Concern about the coronavirus among individuals aged 68–71, 2020.

	Concern for oneself	Concern for family and friends	Concern for society
	(1)	(2)	(3)
Age 68–69: ref. category			
Age 70–71	1.666***	1.428**	1.147
	(0.237)	(0.203)	(0.164)
Control variables:	Yes	Yes	Yes
Cut off points	Yes	Yes	Yes
Pseudo *R*^2^	0.025	0.035	0.031
N	726	726	726

Notes: Ordered logit regression. Coefficients displayed as odds ratios. Dependent variables: ’How worried are you about the coronavirus and its consequences for: 1) Yourself? 2) Your family and friends? 3) Society?’ were 1 = Not at all worried, 2 = Not quite worried, 3 = Somewhat worried, and 4 = Very worried. Standard errors in parentheses.

* p<0.10

** p<0.05

*** p<0.01

The results indicate that being 70 years old, compared to 69, significantly increased the likelihood of experiencing COVID-related worry and anxiety. This suggests that age-specific messaging about COVID-19 vulnerability may have contributed to heightened levels of concern among 70-year-olds compared to their 69-year-old counterparts. It appears that individuals aged 69 perceived themselves as less vulnerable, possibly because they had not yet reached the age group designated as high risk.

As a sensitivity check, we ran all regressions using both logit and OLS models, which yielded similar results.

## Discussion

Effective health risk communication depends not only on disseminating accurate information but also on how different groups perceive the message. It also relies on language preferences and attitudes towards public health interventions [14]. While the transmission of facts is important, research suggests that the message’s perception by different groups can be colored by their beliefs about their level of risk [15]. It is therefore essential to pay attention not only to how the message is disseminated, but how it is perceived by different groups.

The Swedish COVID-19 strategy based on age-specific recommendations, informed by data on infection rates and mortality, consistently highlighted old age as the most significant risk factor for fatal outcomes [1–3, 31]. The aim of this overall communication strategy was clearly to educate and warn the public about age-related variations in severe COVID-19 outcomes in an effort to influence individual behaviour and protect the most vulnerable demographic–the older adults.

Targeted communication and recommendations aimed at specific risk groups can mitigate physical harm and protect those most at risk [16]. However, the process of defining such groups can be complex and may have unintended implications. While it is relatively straightforward to categorize risk based on binary criteria, such as the presence of specific pre-existing conditions (e.g., diabetes), delineating risk based on age is more challenging. The risk of severe illness increases gradually with age, particularly from around 60, 65, or 70 years [31]. The difference in risk between individuals aged 69 and 70 is minimal, much like the difference between individuals one year apart within the same decade (e.g., 68 versus 69-year-olds or 70 versus 71-year-olds). Moreover, older individuals represent a highly heterogeneous group with substantial variability in underlying health conditions, health histories, life experiences, genetics, lifestyles, and overall aging processes [8, 32, 33].

Our study emphasizes that the impact of risk group communication depends on the careful design of messages and can influence how different groups perceive and respond to risks. While the strategy successfully targeted the most vulnerable group, it also inadvertently excluded highly similar risk groups, such as those just one or two years younger. If the definition of a risk group results in individuals near the threshold of the classification perceiving themselves and their health vastly differently–despite negligible actual differences in risk–the intended objective of employing risk group communication can inadvertently backfire.

Our findings reveal the need to consider affected and excluded groups when crafting health risk communication strategies. The way risk groups are defined and communicated can influence self-perception, health concerns, and levels of anxiety, irrespective of actual risk. Alongside the adverse effects that may result from stricter recommendations, ageism and stigma may also act as compounding factors to the adverse effects of being categorized as belonging to a high-risk group [cf. 8–13].

Consequently, the use of risk group communication strategies should be approached with caution, with consideration given to the multifaceted implications it may have on individual perceptions and societal dynamics. This study thereby underscores the critical role of carefully designed risk communication in preventing unnecessary negative consequences, with implications for individual and societal health beyond the context of the pandemic.

## Conclusion

During the early stages of the Covid-19 pandemic, the Swedish Public Health Agency focused its recommendations on individuals aged 70 years and above, who were deemed to be at the highest risk of severe illness from the virus. Starting from March 16, recommendations were issued for this age group, urging them to minimize social interactions, with further age-specific directives issued in the subsequent critical months. These recommendations were in place until October 22, when they were abandoned due to the recognition of adverse consequences such as isolation, lack of social context, and frustration.

While prevailing research has underscored age as a prominent risk factor for severe COVID-19 outcomes, less consideration has been given to the potential impact of being classified into a high-risk group on individuals’ overall health perceptions. Our study addresses this gap by examining disparities in perceived health status and virus-related concerns among individuals aged 69–70 (and 68–71) in Sweden. Drawing on data from 2019 (before the pandemic) and 2020 (during the pandemic), our results indicate a notable divergence, with 70-year-olds reporting a lower perceived health status compared to their 69-year-old counterparts in 2020, but not in 2019. Furthermore, 70-year-olds also expressed higher COVID-19-related concern than 69-year-olds in 2020. This discrepancy suggests that the Swedish COVID-19 strategy, tailored to safeguard those aged 70 and above, may have influenced perceptions of health within this demographic.

Our results suggest that public health strategies, while well-intended, can have unintended consequences. Tailored health communication strategies therefore need to be carefully developed to avoid such negative consequences. Risk group communication based on age may inadvertently exclude similar high-risk individuals, such as those close to the classification threshold. It may also have unintended negative effects on the targeted individuals’ health perceptions, making individuals perceive their overall health as worse than before or worse than it is. Our study also calls for future research to examine whether these disparities persist long after the immediate crisis of the pandemic or diminish over time and revert to pre-pandemic levels relatively quickly.

## Supporting information

S1 File (PDF)
