# Supplementary material for: The impact of health risk communication on self-perceived health and worry of targeted groups: Lessons from the Swedish COVID-19 response
Source: PLoS One. 2025 Jan 17;20(1):e0311596. doi: 10.1371/journal.pone.0311596 (PMC11741659; doi:10.1371/journal.pone.0311596)
Supplement: S1 File — (PDF) [file pone.0311596.s001.pdf]

S1 File

Table S1: Summary statistics for individuals aged 68-71 for the years 2019 and 2020.

Table S2. Concern about the coronavirus among individuals aged 69-70, 2020.

Table S3. Concern about the coronavirus among individuals aged 68-71, 2020.

Table S1: Summary statistics for individuals aged 68-71 for the years 2019 and 2020.

	2019		2020			
	Mean	Sd	Mean	Sd	Min	Max
Dependent variables:						
Subjective health	7.633	1.978	7.494	2.124	0	10
COVID-19: Concern for oneself	.	.	2.674	0.812	1	4
COVID-19: Concern for family	.	.	2.953	0.811	1	4
COVID-19: Concern for the society	.	.	3.014	0.756	1	4
Independent variables:						
Age	69.575	1.137	69.478	1.094	68	71
Household income	4.984	2.567	5.017	2.645	1	12
Higher education	0.294	0.456	0.373	0.484	0	1
Female	0.474	0.500	0.504	0.500	0	1
Villa/ townhouse	0.655	0.476	0.580	0.494	0	1
Single	0.142	0.349	0.180	0.385	0	1
In a relationship	0.039	0.195	0.039	0.193	0	1
Cohabitation	0.114	0.318	0.135	0.342	0	1
Married / Registered partnership	0.640	0.480	0.606	0.489	0	1
Widow / Widower	0.064	0.246	0.040	0.196	0	1
Rural area	0.180	0.384	0.178	0.383	0	1
Smaller agglomeration	0.209	0.407	0.219	0.414	0	1
City or larger agglomeration	0.469	0.499	0.460	0.499	0	1
Stockholm/ Gothenburg/ Malmo	0.142	0.349	0.143	0.351	0	1
Month: September	0.621	0.485	0.669	0.471	0	1
Month: October	0.308	0.462	0.262	0.440	0	1
Month: November	0.050	0.217	0.052	0.223	0	1
Month: Dec/Jan	0.020	0.142	0.017	0.128	0	1
Observations	684		726			

Notes: Summary statistics year 2019 (column 1 and 2), and year 2020 (column 3 and 4).

Table S2. Concern about the coronavirus among individuals aged 69-70, 2020.

	Concern for oneself (1)	Concern for family and friends (2)	Concern for society (3)
Age 69: ref. category			
Age 70	2.053***	1.542**	1.119
	(0.414)	(0.309)	(0.225)
Household income	0.880***	0.864***	0.899**
	(0.038)	(0.038)	(0.040)
Higher education	0.714	0.596**	0.681*
	(0.155)	(0.129)	(0.151)
Female	1.613**	1.589**	1.729***
	(0.339)	(0.332)	(0.366)
Villa/ townhouse	1.072	0.980	0.773
	(0.249)	(0.229)	(0.184)
Single: ref. category			
In a relationship	0.568	1.002	0.518
	(0.341)	(0.601)	(0.312)
Cohabitation	1.204	0.919	0.852
	(0.462)	(0.345)	(0.335)
Married/ Registered partnership	1.156	1.096	0.860
	(0.368)	(0.346)	(0.275)
Widow/ Widower	0.739	0.241**	0.711
	(0.433)	(0.144)	(0.444)
Rural area: ref. category			
Smaller agglomeration	0.972	0.609	1.236
	(0.313)	(0.198)	(0.403)
City or larger agglomeration	1.184	1.249	1.621*
	(0.330)	(0.344)	(0.459)
Stockholm/ Gothenburg/ Malmo	1.982*	1.980*	2.646**
	(0.781)	(0.768)	(1.072)
Month: September: ref. category			
Month: October	1.512*	1.475*	1.468
	(0.350)	(0.342)	(0.350)
Month: November	2.666**	2.750**	3.044**
	(1.279)	(1.298)	(1.503)
Month: Dec/Jan	6.070	3.914	0.696
	(8.265)	(5.671)	(1.195)
Cut off points	Yes	Yes	Yes
Pseudo R^2	0.056	0.065	0.056
N	383	383	383

Notes: Ordered logit regression. Coefficients displayed as odds ratios. Dependent variables: 'How worried are you about the coronavirus and its consequences for: 1) Yourself? 2) Your family and friends? 3) Society?' were 1 = Not at all worried, 2 = Not quite worried, 3 = Somewhat worried, and 4 = Very worried. Standard errors in parentheses.
* p<0.10, ** p<0.05, *** p<0.01

Table S3. Concern about the coronavirus among individuals aged 68-71, 2020.

	Concern for oneself (1)	Concern for family and friends (2)	Concern for society (3)
Age 68-69: ref. category			
Age 70-71	1.666***	1.428**	1.147
	(0.237)	(0.203)	(0.164)
Household income	0.937**	0.946*	0.943*
	(0.030)	(0.030)	(0.031)
Higher education	0.797	0.674**	0.638***
	(0.123)	(0.104)	(0.100)
Female	1.470***	1.456**	1.563***
	(0.218)	(0.216)	(0.234)
Villa/ townhouse	1.017	0.822	0.945
	(0.170)	(0.138)	(0.160)
Single: ref. category			
In a relationship	0.835	1.703	1.001
	(0.338)	(0.683)	(0.396)
Cohabitation	0.953	0.911	0.803
	(0.251)	(0.239)	(0.215)
Married/ Registered partnership	1.110	1.210	1.051
	(0.236)	(0.258)	(0.227)
Widow/ Widower	0.618	0.270***	0.488*
	(0.247)	(0.106)	(0.195)
Rural area: ref. category			
Smaller agglomeration	0.861	0.726	0.887
	(0.194)	(0.162)	(0.203)
City or larger agglomeration	1.179	1.072	1.189
	(0.244)	(0.220)	(0.249)
Stockholm/ Gothenburg/ Malmo	1.437	1.111	1.480
	(0.405)	(0.309)	(0.430)
Month: September: ref. category			
Month: October	1.334*	1.286	1.443**
	(0.218)	(0.210)	(0.240)
Month: November	1.320	1.575	2.391***
	(0.429)	(0.514)	(0.790)
Month: Dec/Jan	2.387	6.025***	3.161**
	(1.274)	(3.869)	(1.824)
Cut off points	Yes	Yes	Yes
Pseudo R^2	0.025	0.035	0.031
N	726	726	726

Notes: Ordered logit regression. Coefficients displayed as odds ratios. Dependent variables: 'How worried are you about the coronavirus and its consequences for: 1) Yourself? 2) Your family and friends? 3) Society?' were 1 = Not at all worried, 2 = Not quite worried, 3 = Somewhat worried, and 4 = Very worried. Standard errors in parentheses.
* p<0.10, ** p<0.05, *** p<0.01
